# Hyperosmolality regulates UT‐A6 urea transporter expression in the Caco‐2 cell line

**DOI:** 10.14814/phy2.12984

**Published:** 2016-09-26

**Authors:** Alison McGrane, Gavin Stewart

**Affiliations:** ^1^ School of Biology & Environmental ScienceScience Centre WestUniversity College DublinDublinIreland

**Keywords:** Caco‐2, expression, hUT‐A6, hyperosmolality

## Abstract

Gastrointestinal facilitative urea transporters play a significant role in the urea nitrogen salvaging process, which supports the symbiotic relationship between mammals and their gut microbial populations. UT‐A6 urea transporters have been previously reported in the human gastrointestinal tract, specifically in the colon. As renal UT‐A transporters can be regulated by external osmolality, this study investigated whether UT‐A6 expression could also be regulated in this manner. Initial end‐point RT‐PCR experiments confirmed UT‐A6 expression along the human gastrointestinal tract (colon > small intestine ≫ stomach) and also in the Caco‐2 intestinal cell line. Using Caco‐2 cells exposed for 24 hours to changed external osmotic conditions (from 350 to 250, 500, or 600 mOsm), end‐point PCR suggested UT‐A6 expression increased in hyperosmotic conditions. Using quantitative PCR, it was confirmed that 24 h exposure to 600 mOsm stimulated a significant ~15‐fold increase in UT‐A6 expression (*P* < 0.001, *N* = 5, ANOVA). Finally, inhibitory experiments suggested that protein kinase C and calcium were involved in this hyperosmotic‐stimulated regulatory pathway. In conclusion, these data demonstrated UT‐A6 expression was indeed regulated by external osmolality. The physiological significance of this regulatory process upon gastrointestinal urea transport has yet to be determined.

## Introduction

Mammalian facilitative urea transporters (UTs), encoded by either Slc14a1 (UT‐B) or Slc14a2 (UT‐A) genes, facilitate the movement of urea across plasma cell membranes (Stewart 2011). In the kidney, renal UTs play a crucial role in the urinary concentrating mechanism (Fenton et al. 2004). In contrast, gastrointestinal urea transporters play a significant role in the urea nitrogen salvaging process, which supports the symbiotic relationship between mammals and the microbial populations within their gastrointestinal tract (Stewart and Smith 2005).

**Figure 1:**
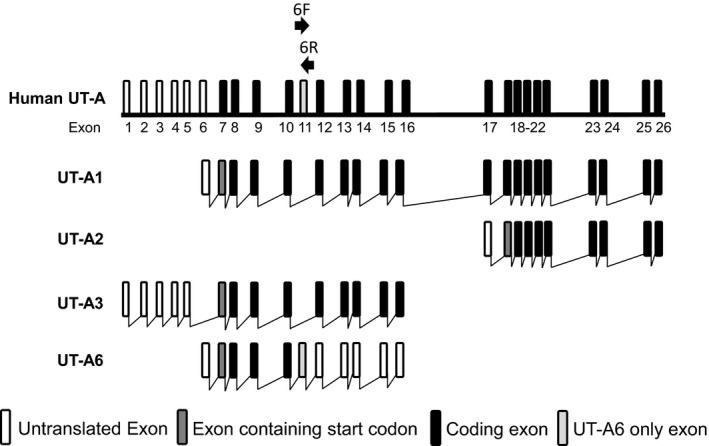
Schematic diagram representing the basic structure of the human *SLC14A2* (UT‐A) gene. It should be noted that exon 11 is only included in UT‐A6 and not in any of the other UT‐A isoforms. Labeled arrows show the location and direction of the forward (6F) and reverse (6R) primers used to amplify UT‐A6 in this study. [Diagram adapted from Smith and Fenton 2006.]

Most previous studies investigating gastrointestinal UTs have focused on UT‐B transporters. For example, functional UT‐B urea transporter proteins have been detected in bovine rumen (Stewart et al. 2005), human colon (Collins et al. 2010), rat colon (Collins et al. 2011), and also in the Caco‐2 intestinal cell line (Inoue et al. 2004). However, UT‐A6 is a unique UT‐A urea transporter that has also been detected in the human colon (Smith et al. 2004). The UT‐A6 transcript contains a novel 129‐bp exon not found in other UT‐A transcripts (Smith et al. 2004), originally termed exon 5a (Smith et al. 2004), but now known to be exon 11 within the human UT‐A gene (Smith and Fenton 2006) (see Fig. 1). The UT‐A6 transcript actually encodes a 235 amino acid protein, making it the smallest member of the UT‐A family (Smith et al. 2004). Northern blot analysis showed UT‐A6 to be expressed in human colon, but not stomach or small intestine (Smith et al. 2004).

At present, very little is understood regarding the physiological role or regulation of the UT‐A6 urea transporter. However, expression of renal UT‐A transporters is widely reported to be regulated by osmolality in various mammals, such as mice (Fenton et al. 2002) and rats (Nakayama et al. 2000). The aim of this study was therefore to investigate whether changes in external osmolality could regulate UT‐A6 expression using the Caco‐2 cell line.

## Methods

### Maintenance of Caco‐2 cell line

Human epithelial colorectal adenocarcinoma Caco‐2 cells (HTB‐37) were purchased (ATCC, Manassas, VA) and were cultured in Dulbecco's Modified Eagle Medium (DMEM) with a high d‐glucose content (4.5 g/L), containing l‐glutamine, and supplemented with 10% heat‐inactivated fetal bovine serum (Life Technologies, Dublin, Ireland). Once cells reached 90% confluency, they were subcultured in a 1:6 dilution every 5 days. Media were removed and replaced with fresh media every 2–3 days. Cells were incubated at 37°C with 5% CO_2_. For regulatory experiments, cells which had reached 90% confluency were exposed to media containing additional NaCl, mannitol, or butyrate for 24 h prior to RNA extraction. Osmolalities of different experimental media were confirmed using an Osmomat 030 osmometer (Gonotec, Germany) and measurements (mean ± standard error) were as follows: “250 mOsm” = 246 ± 22 mOsm (*N* = 4); Control “350 mOsm” = 354 ± 13 mOsm (*N* = 5); “500 mOsm” = 502 ± 9 mOsm (*N* = 3); “600 mOsm” with NaCl = 595 ± 30 mOsm (*N* = 5); and “600 mOsm” with mannitol = 634 ± 15 mOsm (*N* = 4).

### RNA preparation

Caco‐2 cells were grown in 75 mm^2^ plastic flasks and total RNA isolated from confluent cells using a standard RNA extraction protocol using RNA Stat (AMS Biotechnology, Abingdon, UK), BCP (1‐Bromo‐3‐chloropropane), isopropanol, and ethanol. Total RNA samples were DNAase (Life Technologies) treated for 25 min at 37°C and then quantified using a ND‐1000 Nanodrop Spectrophotometer (Labtech International, Batam, Indonesia). Using sterile distilled H_2_O, RNA samples were diluted (to concentration of 250 ng/μL) and stored at −80°C until use.

### End‐point RT‐PCR

Using purchased human tissue RNA samples (AMS Biotechnology) or the prepared Caco‐2 total RNA samples, cDNA preparation was performed using a SensiFast cDNA synthesis kit (Bioline, London, UK). Resulting cDNA samples underwent PCR amplification with a Go‐*Taq* polymerase enzyme (Promega, Kilkenny, Ireland), using UT‐A6, AQP3, or actin primers (see Table 1 for primer sequences).

**Table 1:** Sequences of all end‐point PCR primer sets, plus expected product sizes

Primer	Sequence	Product size
UT‐A6 forward	5′‐GATGGAGACGGATTTTTAACTGGAGTA‐3′	128 bp
UT‐A6 reverse	5′‐GCATGTTCATGGATATTCACTCTAATCT‐3′	
AlUT‐A6 forward	5′‐GGATGGAGACGGATTTTAACTGGAG‐3′	136 bp
AlUT‐A6 reverse	5′‐CCTGTGGCATGTTCATGGATATCACTC‐3′	
AQP3 forward	5′‐GGGAGCCTTCTTGGGTGCTG‐3′	286 bp
AQP3 reverse	5′‐GGAGGTGCCAATGACCAGGAC‐3′	
Actin forward	5′‐GTGCTGTCTGGCACCACCAT‐3′	514 bp
Actin reverse	5′‐CCTGTAACAACGCATCTCATAT‐3′	

Cycling parameters were initial denaturation at 94°C for 2 min, followed by 30 or 35 cycles at 94°C for 30 sec, 55°C or 60°C for 30 sec, and 72°C for 30 sec. The final extension was at 72°C for 5 min. Identity of PCR products was confirmed through direct sequencing (Eurofins MWG, Operon, Germany).

### Quantitative PCR

PCR reactions were prepared in MicroAmp Fast Optical 96‐well reaction plates using Fast SYBR Green Master mix (Applied Biosystems, Dublin, Ireland) and qPCR primers (see Table**** 2). Plates were sealed with MicroAmp Optical Adhesive film (Applied Biosystems) and experiments performed using a qPCR machine (ViiA 7 Real‐Time PCR System, Life Technologies). The ViiA 7 Software, Version 1.2.3, was used for qPCR analysis.

**Table 2:** Sequences of quantitative PCR primers

Primer	Sequence
q1hUT‐A6_for	5′‐TGGAGTAAGAGTGGATGCTGGAA‐3′
q1hUT‐A6_rev	5′‐TCACTCTAATCTAAACACTGTCATCTCTG‐3′
q3hActin‐for	5′‐CCACCCCACTTCTCTCTAAGGA‐3′
q3hActin‐rev	5′‐ACCTCCCCTGTGTGGACTTG‐3′

## Results

**Figure 2:**
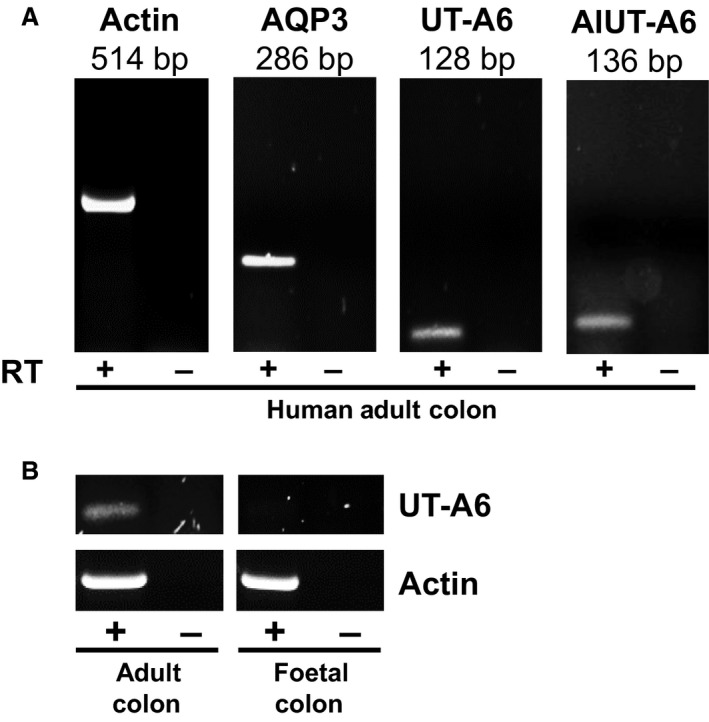
End‐point PCR experiments investigating UT‐A6 expression in human colon. (A) Using cDNA derived from pooled total RNA adult colon samples (*N* = 5), strong signals were detected for actin (514 bp) and AQP3 (286 bp). In contrast, weaker signals were detect for UT‐A6 using two separate sets of primers designed against the UT‐A6‐specific exon 11 (128 and 136 bp signals). These signals were confirmed as UT‐A6 by sequencing of the PCR products. (B) Using cDNA derived from total RNA adult and fetal colon samples (each *N* = 1), a weak UT‐A6 signal was again detected in adult colon, but not in fetal colon. Key: RT = reverse transcriptase; + = RT present; − = RT absent.

Initial end‐point PCR experiments were performed to investigate colonic UT‐A6 expression using two different sets of primers, one previously utilized set (i.e., “UT‐A6”) (Smith et al. 2004) and another alternative set (i.e., “AlUT‐A6”). All four of these primers were targeted to the UT‐A6‐specific exon 11 (see Fig. 1). Using cDNA derived from adult colon total RNA, pooled from five donors, signals for UT‐A6 were detected at sizes of 128 and 136 bp as expected (see Fig. 2A). In contrast, much stronger signals were detected for actin (514 bp) and, more importantly, for another colonic membrane transporter aquaporin 3, AQP3 (286 bp). Interestingly, additional experiments showed that UT‐A6 did not appear to be expressed in human fetal colon (see Fig. 2B).

**Figure 3:**
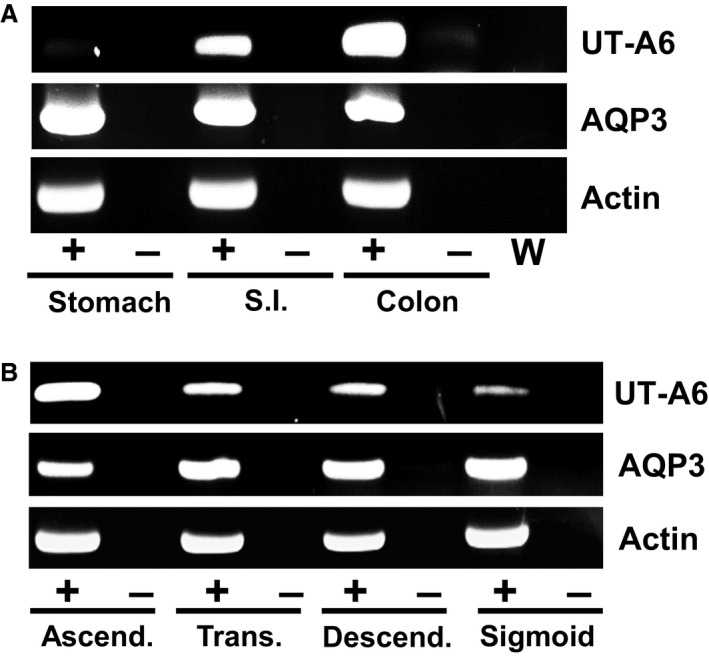
End‐point PCR experiments investigating UT‐A6 expression in the human gastrointestinal tract. (A) Using cDNA derived from mRNA samples (each *N* = 1), strong signals were detected for actin and AQP3 in all gastrointestinal tissues. In contrast, 128 bp UT‐A6 signals were varied, with a clear expression pattern showing colon > small intestine ≫ stomach. (B) Using cDNA derived from total RNA colonic samples (each *N* = 1), UT‐A6 signals were again varied with the strongest signal in ascending colon and the weakest signal in sigmoid colon. No such differences were observed for either actin or AQP3. Key: RT = reverse transcriptase; + = RT present; − = RT absent; W = water control.

Next, end‐point PCR experiments were performed to investigate UT‐A6 expression along the length of the human gastrointestinal tract. Using cDNA derived from high‐quality mRNA samples, strong UT‐A6 128 bp signals were detected in human colon and small intestine samples, but not in the stomach (see Fig. 3A). Furthermore, variable UT‐A6 signals were present in different regions of the human colon, with the strongest signal in the ascending colon and the weakest in the sigmoid colon (see Fig. 3B). Importantly, strong AQP3 and actin signals were detected in all of these gastrointestinal samples.

**Figure 4:**
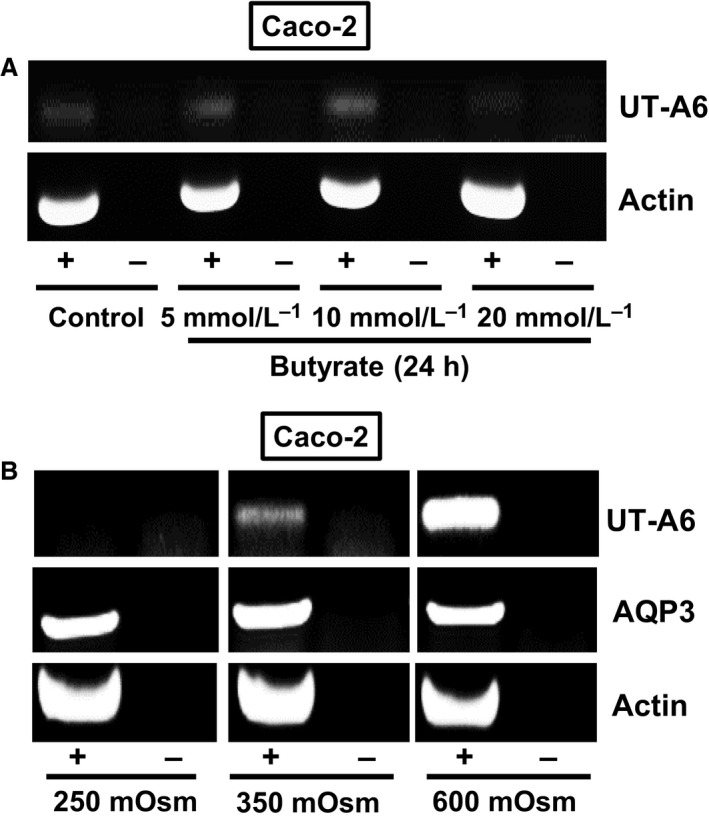
End‐point PCR experiments investigating UT‐A6 expression in the Caco‐2 cell line. (A) Using cDNA derived from total RNA samples (each *N* = 1), only very weak UT‐A6 signals were detected. These UT‐A6 signals were unaffected by 24 h incubation in different concentrations of butyrate. (B) In contrast, UT‐A6 signals varied with changing the osmolality of the culture media for 24 h prior to RNA preparation. No UT‐A6 signal was detected in the 250 mOsm samples, a weak signal was present with 350 mOsm (i.e., normal media), and a strong signal obtained with 600 mOsm. In contrast, no changes were observed in either actin or AQP3 signals. Key: RT = reverse transcriptase; + = RT present; − = RT absent.

As UT‐A6 expression varied along the length of the human gastrointestinal tract, the next set of end‐point PCR experiments investigated the regulatory process involved. Using cDNA derived from various Caco‐2 total RNA samples, it was clearly observed that although weak UT‐A6 could be detected, this signal was not affected by 24 h incubation in various concentrations of the short‐chain fatty acid butyrate (see Fig. 4A). Similarly, exposure for 24 h to media containing a physiologically relevant concentration of urea (i.e., 10 mmol/L) had no effect on UT‐A6 expression (data not shown). However, alterations in the osmolality of the culture media had a noticeable effect on UT‐A6 expression (see Fig. 4B). The UT‐A6 signal was significantly stronger in Caco‐2 cells exposed to hyperosmotic conditions for 24 h (i.e., increased from a control level of 350–600 mOsm using additional NaCl). In contrast, when external osmolality was reduced to 250 mOsm, by diluting control media with sterile water, there was no longer any UT‐A6 signal detected (see Fig. 4B). Once again, no such differences were observed for AQP3 and actin, as strong signals were present in each sample.

As simple end‐point PCR is not a reliable method to precisely analyze changes in RNA expression, further experiments were performed using quantitative PCR. Using primers sets designed against UT‐A6 and actin, quantitative PCR was performed using multiple RNA samples from Caco‐2 cells exposed for 24 h to 250, 350, 500, or 600 mOsm. As can be seen in Table 3, compared to levels in control conditions of 350 mOsm, significant increases in UT‐A6 expression were confirmed in cells exposed to either 500 mOsm (*P* = 0.0033, *N* = 3, ANOVA) or 600 mOsm (*P* = 0.0003, *N* = 5, ANOVA). This change with 600 mOsm conditions actually equates to ~15‐fold increase in UT‐A6 expression. For the 250 mOsm samples, while the expected lower than control UT‐A6 expression was observed quantitative PCR showed that there was no significant change (*P* = 0.7590, *N* = 4, ANOVA).

**Table 3:** qPCR data for Caco‐2 cells exposed for 24 h to media of varying osmolality. Primers for both UT‐A6 and actin were used and the results compared. Data are mean ± standard deviation. Statistical analysis (ANOVA) is relative to control media (350 mOsm)

External osmolality (mOsm)	UT‐A6 average C_*T*_	Actin average C_*T*_	UT‐A6 – Actin ΔC_*T*_	*P*‐value versus 350
250 *N* = 4	32.7 ± 0.5	15.5 ± 1.1	17.3 ± 1.1	0.7590
350 *N* = 5	31.0 ± 0.7	15.7 ± 1.6	15.3 ± 1.2	–
500 *N* = 3	28.3 ± 0.8	16.4 ± 1.1	11.8 ± 1.6	0.0033
600 *N* = 5	28.4 ± 0.6	16.9 ± 1.3	11.5 ± 0.9	0.0003

**Figure 5:**
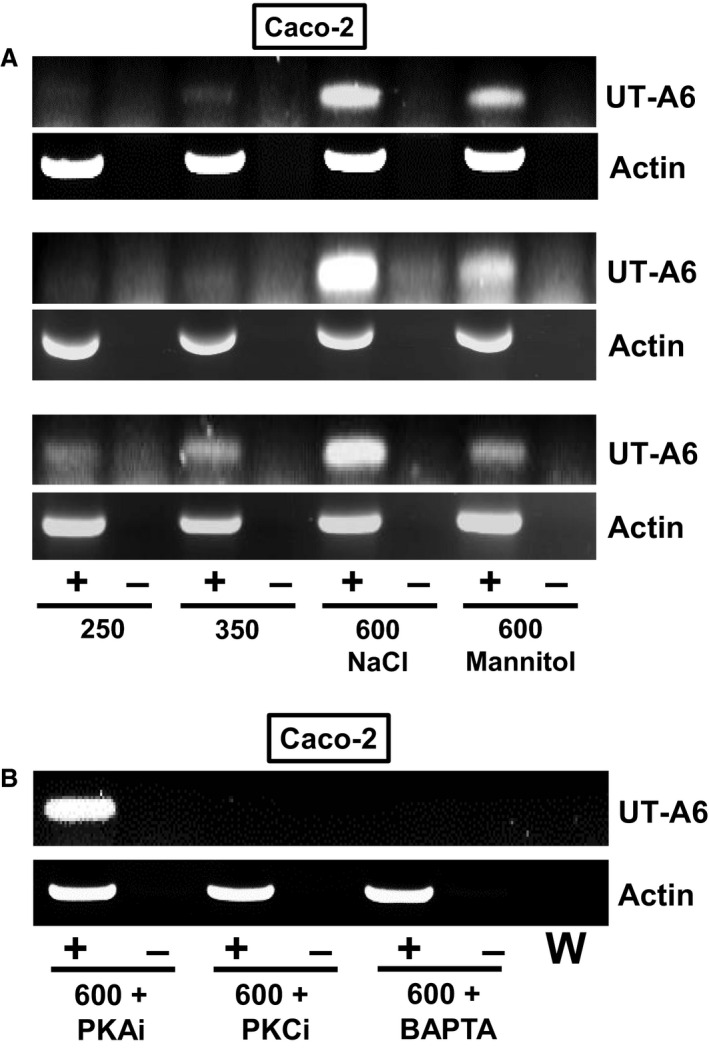
End‐point PCR experiments further investigating UT‐A6 expression in the Caco‐2 cell line. (A) Increased UT‐A6 signals were detected with hyperosmotic media (i.e., 600 mOsm due to an additional osmolyte), with greater increases observed with NaCl when compared to mannitol. (B) Addition of either a protein kinase C inhibitor or an internal calcium chelator (i.e., BAPTA‐AM) to the culture media totally ablated the UT‐A6 signal in 600 mOsm hyperosmotic conditions. No such effect was observed with the addition of a protein kinase A inhibitor. Key: RT = reverse transcriptase; + = RT present; − = RT absent; W = water control; PKAi = 0.5μmol/L protein kinase A inhibitor; PKCi = 1 μmol/L calphostin C (i.e., protein kinase C inhibitor); BAPTA = 50 μmol/L BAPTA‐AM.

Lastly, some preliminary end‐point PCR experiments were performed to further investigate the potential mechanism involved in the hyperosmotic stimulation of UT‐A6 expression. Increasing the culture media osmolality to 600 mOsm with mannitol had the same stimulatory effect on UT‐A6 expression as using NaCl (see Fig. 5A), although mannitol did not appear to stimulate UT‐A6 expression to the same extent. In the final experiment, preincubation with Calphostin C (protein kinase C inhibitor) or BAPTA‐AM (a cell permeable calcium chelator) totally ablated UT‐A6 expression, whereas a protein kinase A inhibitor had no effect (see Fig. 5B).

## Discussion

Over a decade ago, UT‐A6 was characterized as a unique urea transporter located in human colon (Smith et al. 2004). Little else has been discovered regarding this transporter, therefore the aim of this study was to investigate the regulation of UT‐A6 expression.

As expected, initial end‐point PCR experiments confirmed that UT‐A6 was weakly expressed in the human adult colon (see Fig. 2A). Interestingly, it was not detected in human fetal colon (see Fig. 2B), suggesting that UT‐A6 expression was regulated by the contents of the gastrointestinal tract. Further end‐point PCR experiments produced an expression pattern of colon > small intestine ≫ stomach (see Fig. 3A), with the strongest colonic signal being located in the ascending colon (see Fig. 3B). These data are in complete agreement with the Northern blot analysis reported in the original UT‐A6 study (Smith et al. 2004). These findings also agree with our previous investigation showing increased glycosylated UT‐B protein and trans‐epithelial urea transport in the right (i.e., ascending) colon (Collins et al. 2010). These data suggest that UT‐A6 could play a role in trans‐epithelial urea transport, in combination with UT‐B transporters, particularly in the ascending colon.

In order to investigate regulation of UT‐A6 expression, further experiments were performed to first confirm it was expressed in the Caco‐2 cell line, as these cells had previously been shown to be a relevant model for gastrointestinal UT‐B transporters (Inoue et al. 2004). Our data confirmed that UT‐A6 was weakly expressed in Caco‐2 cells (Fig. 4A), but that it was not regulated by exposure to butyrate – unlike some other membrane transporters involved in mammalian‐microbial symbiotic gastrointestinal functions, for example, the short‐chain fatty acid transporter MCT1 (Borthakur et al. 2008). In contrast, exposing Caco‐2 cells for 24 h to hyperosmotic conditions (i.e., 600 mOsm) significantly increased UT‐A6 expression (see Fig. 4B). Importantly, the increase in UT‐A6 expression was confirmed by quantitative PCR analysis to be statistically significant (*P* < 0.001, *N* = 5, ANOVA) (see Table 3). These findings are in agreement with previous studies, in which an increase to 600 mOsm for 24 h has been shown to upregulate UT‐A gene expression in rats (Nakayama et al. 2000) and mice (Fenton et al. 2002). A key novel finding of this study was that the stimulation of UT‐A6 expression by hyperosmotic conditions, which occurred with both NaCl and mannitol (see Fig. 5A), was prevented by either the inhibition of protein kinase C or a reduction in intracellular calcium levels (see Fig. 5B).

Previous studies have shown that acute exposure to hyperosmolality, using solutions containing either additional NaCl or additional mannitol, increased urea permeability of the rat terminal inner medullary collecting duct (Sands and Schrader 1991). After the subsequent discovery of facilitative urea transporters, it was later shown that this increased transport occurred through a regulatory process involving protein kinase C‐mediated phosphorylation of UT‐A1 and UT‐A3 urea transporters (Wang et al. 2010), specifically the calcium‐dependent protein kinase C‐alpha isoform (Klein et al. 2012). Interestingly, both protein kinase C and calmodulin – a calcium‐dependent protein – were also shown to be involved in the regulation of a mouse renal urea transporter, mUT‐A3 (Stewart et al. 2009). Crucially, while all these studies demonstrate the role for protein kinase C and calcium in the acute regulation of UT‐A urea transporter protein function, this study is the first to implicate these two factors in the chronic regulation of UT‐A transcription. Interestingly, protein kinase C isoforms have previously been reported to be involved with regulation of various intestinal transporters, such as MCT1 (Saksena et al. 2009) and sodium–hydrogen exchanger NHE3 (Alrefai et al. 2001). The potential mechanism by which protein kinase C and calcium could be influencing these genomic events is not yet understood, but should be the topic of detailed future investigations. In addition, given the intestinal context of these findings regarding UT‐A6 regulation, further studies are required to determine whether high levels of substrates found in the colonic lumen (e.g., acetate, propionate) can also elicit this hyperosmotic‐induced upregulation of UT‐A6 expression.

In conclusion, this study has shown that intestinal UT‐A6 transporter expression can be regulated by hyperosmolality, in a manner similar to that previously described for renal UT‐A transporters. Future experiments should now focus on the detection, cellular localization, and functional investigation of UT‐A6 protein. It will only be through such studies that we will determine the functional relevance of UT‐A6 transporters and hence understand their physiological significance within the human colon.

## Conflict of Interest

None declared.

## References

[phy212984-bib-0001] Alrefai, W. A. , B. Scaglione‐Seweel , S. Tyagi , L. Wartman , T. A. Brasitus , K. Ramaswamy , et al. 2001 Differential regulation of the expression of Na(+)/H(+) exchanger isoform NHE3 by PKC‐alpha in Caco‐2 cells. Am. J. Physiol. Cell Physiol. 281:C1551–C1558. 11600418 10.1152/ajpcell.2001.281.5.C1551

[phy212984-bib-0002] Borthakur, A. , S. Saksena , R. K. Gill , W. A. Alrefai , K. Ramaswamy , and P. K. Dudeja . 2008 Regulation of monocarboxylate transporter 1 (MCT1) promoter by butyrate in human intestinal epithelial cells: involvement of NF‐kappaB pathway. J. Cell. Biochem. 103:1452–1463. 17786924 10.1002/jcb.21532 PMC2673490

[phy212984-bib-0003] Collins, D. , D. C. Winter , A. M. Hogan , L. Schirmer , A. W. Baird , and G. S. Stewart . 2010 Differential protein abundance and function of UT‐B urea transporters in human colon. Am. J. Physiol. Gastrointest. Liver Physiol. 298:G345–G351. 19926813 10.1152/ajpgi.00405.2009 PMC3774180

[phy212984-bib-0004] Collins, D. , C. Walpole , E. Ryan , D. Winter , A. Baird , and G. Stewart . 2011 UT‐B1 mediates trans‐epithelial urea flux in the rat gastrointestinal tract. J. Membr. Biol. 239:123–130. 21127847 10.1007/s00232-010-9331-9

[phy212984-bib-0005] Fenton, R. A. , C. A. Cottingham , G. S. Stewart , A. Howorth , J. A. Hewitt , and C. P. Smith . 2002 Structure and characterization of the mouse UT‐A gene (Slc14a2). Am. J. Physiol. Renal Physiol. 282:F630–F638. 11880324 10.1152/ajprenal.00264.2001

[phy212984-bib-0006] Fenton, R. A. , C. Chou , G. S. Stewart , C. P. Smith , and M. A. Knepper . 2004 Urinary concentrating defect in mice with selective deletion of phloretin‐sensitive urea transporters in the renal collecting duct. Proc. Natl. Acad. Sci. U. S. A. 101:7469–7474. 15123796 10.1073/pnas.0401704101 PMC409942

[phy212984-bib-0007] Inoue, H. , S. D. Jackson , T. Vikulina , J. D. Klein , K. Tomita , and S. M. Bagnasco . 2004 Identification and characterization of a Kidd antigen/UT‐B urea transporter expressed in human colon. Am. J. Physiol. Cell Physiol. 287:C30–C35. 14985236 10.1152/ajpcell.00443.2003

[phy212984-bib-0008] Klein, J. D. , C. F. Martin , K. J. Kent , and J. M. Sands . 2012 Protein kinase C alpha mediates hypertonicity‐stimulated increase in urea transporter phosphorylation in the inner medullary collecting duct. Am. J. Physiol. Renal Physiol. 302:F1098–F1103. 22301620 10.1152/ajprenal.00664.2011 PMC3362171

[phy212984-bib-0009] Nakayama, Y. , T. Peng , J. M. Sands , and S. M. Bagnasco . 2000 The TonE/TonEBP pathway mediates tonicity‐responsive regulation of UT‐A urea transporter expression. J. Biol. Chem. 275:38275–3280. 10995747 10.1074/jbc.M004678200

[phy212984-bib-0010] Saksena, S. , A. Dwivedi , R. K. Gill , A. Singla , W. A. Alrefai , J. Malakooti , et al. 2009 PKC‐dependent stimulation of the human MCT1 promoter involves transcription factor AP2. Am. J. Physiol. Gastrointest. Liver Physiol. 296:G275–G283. 19033536 10.1152/ajpgi.90503.2008 PMC2643915

[phy212984-bib-0011] Sands, J. M. , and D. C. Schrader . 1991 An independent effect of osmolality on urea transport in rat terminal IMCDs. J. Clin. Invest. 88:137–142. 1905326 10.1172/JCI115269 PMC296013

[phy212984-bib-0012] Smith, C. P. , and R. A. Fenton . 2006 Genomic organization of the mammalian *SLC14A2* urea transporter genes. J. Membr. Biol. 212:109–117. 17264986 10.1007/s00232-006-0870-z

[phy212984-bib-0013] Smith, C. P. , E. A. Potter , R. A. Fenton , and G. S. Stewart . 2004 Characterization of a human colonic cDNA encoding a structurally novel urea transporter, UT‐A6. Am. J. Physiol. Cell Physiol. 287:C1087–C1093. 15189812 10.1152/ajpcell.00363.2003

[phy212984-bib-0014] Stewart, G. S. 2011 The emerging physiological roles of the SLC14A family of urea transporters. Br. J. Pharmacol. 164:1780–1792. 21449978 10.1111/j.1476-5381.2011.01377.x PMC3246703

[phy212984-bib-0015] Stewart, G. S. , and C. P. Smith . 2005 Urea nitrogen salvage mechanisms and their relevance to ruminants, non‐ruminants and man. Nutr. Res. Rev. 18:49–62. 19079894 10.1079/NRR200498

[phy212984-bib-0016] Stewart, G. S. , C. Graham , S. Cattell , T. P. L. Smith , N. L. Simmons , and C. P. Smith . 2005 UT‐B is expressed in bovine rumen: potential role in ruminal urea transport. Am. J. Physiol. Regul. Integr. Comp. Physiol. 289:R605–R612. 15845882 10.1152/ajpregu.00127.2005

[phy212984-bib-0017] Stewart, G. S. , A. Thistlethwaite , H. Lees , G. J. Cooper , and C. P. Smith . 2009 Vasopressin regulation of the renal UT‐A3 urea transporter. Am. J. Physiol. Renal Physiol. 296:F642–F649. 19052101 10.1152/ajprenal.90660.2008

[phy212984-bib-0018] Wang, Y. , J. D. Klein , C. M. Liedtke , and J. M. Sands . 2010 Protein kinase C regulates urea permeability in the rat inner medullary collecting duct. Am. J. Physiol. Renal Physiol. 299:F1401–F1406. 20861079 10.1152/ajprenal.00322.2010 PMC3006311

